# Computer Vision Techniques Demonstrate Robust Orientation Measurement of the Milky Way Despite Image Motion

**DOI:** 10.3390/biomimetics9070375

**Published:** 2024-06-21

**Authors:** Yiting Tao, Asanka Perera, Samuel Teague, Timothy McIntyre, Eric Warrant, Javaan Chahl

**Affiliations:** 1 School of Engineering, University of South Australia, Mawson Lakes, SA 5095, Australia; samuel.teague@mymail.unisa.edu.au (S.T.); timothy.mcintyre@unisa.edu.au (T.M.); javaan.chahl@unisa.edu.au (J.C.); 2 School of Engineering, University of Southern Queensland, Springfield, QLD 4300, Australia; asanka.perera@unisq.edu.au; 3 Defence Science and Technology Group, Platforms Division, Edinburgh, SA 5111, Australia; 4 Lund Vision Group, Department of Biology, University of Lund, SE-221 00 Lund, Sweden; eric.warrant@biol.lu.se

**Keywords:** biomimetic, Milky Way, object detection, orientation, motion blur

## Abstract

Many species rely on celestial cues as a reliable guide for maintaining heading while navigating. In this paper, we propose a method that extracts the Milky Way (MW) shape as an orientation cue in low-light scenarios. We also tested the method on both real and synthetic images and demonstrate that the performance of the method appears to be accurate and reliable to motion blur that might be caused by rotational vibration and stabilisation artefacts. The technique presented achieves an angular accuracy between a minimum of 0.00° and a maximum 0.08° for real night sky images, and between a minimum of 0.22° and a maximum 1.61° for synthetic images. The imaging of the MW is largely unaffected by blur. We speculate that the use of the MW as an orientation cue has evolved because, unlike individual stars, it is resilient to motion blur caused by locomotion.

## 1. Introduction

In the natural world, animals, including insects, have evolved diverse mechanisms to measure their direction during navigation and locomotion [1]. These mechanisms involve the use of sensory cues from the environment, including visual landmarks (e.g., pigeons [2]), celestial cues (e.g., desert ants [3]), magnetic cues (e.g., migratory birds [4]), and wind (e.g., *Drosophila* [5]).

Many species rely on celestial cues such as the sun, the moon, polarised light, the stars, and the Milky Way, with each serving as a reliable guide for maintaining heading [6,7,8,9]. Despite having tiny brains, insects have evolved remarkable sensory mechanisms that allow robust navigation in diverse environments. The ability to forage, migrate, and escape from danger in a straight line through or over complex terrain is crucial for insects.

Amongst the celestial information that can be extracted from the environment for orientation, the MW presents distinct characteristics for navigation that are different from the precise cues provided by stars. Under a clear sky, the MW offers a large, extended celestial landmark, with distinctive features, including a gradient of increasing intensity from north to south. The nocturnal dung beetle, *Scarabaeus satyrus*, has evolved the ability to exploit these features, using the MW for maintaining heading while orienting [10,11] during transit.

The night sky is sprinkled with a multitude of stars of varying intensity that are easily detectable by dark-adapted human observers. However, since the nocturnal beetle’s tiny compound eyes limit the visibility of point sources like stars, the majority of these stars are likely to be too dim to distinguish effectively [10], particularly while in motion.

**Figure 1:**
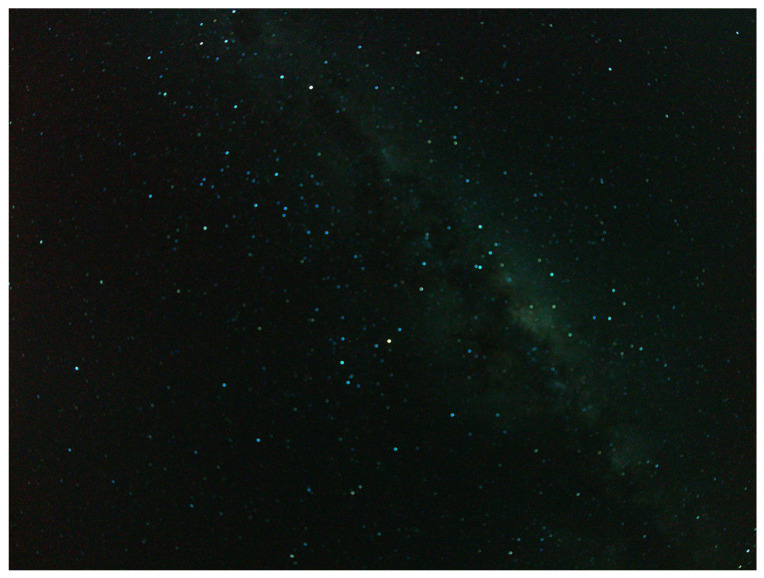
The Milky Way (MW) observed under a rural sky in South Australia.

For insect sensory purposes, the MW is extensive, and it is comparatively low-contrast (Figure 1). However, due to its wide extent, the MW is highly visible to the compound eyes of nocturnal insects, whose typically low F-number optics ensure that the retinal image of the MW is bright. This high optical sensitivity ensures that despite being dim and of low contrast, the MW is easily seen. The large size of the visual feature is useful for compound eyes since high spatial acuity is not required.

### Contribution of This Study

Therefore, this study investigated the influence of motion blur through computer vision algorithms as objective measurement tools and proposes a method for maintaining the orientation performance in low-light scenarios despite high levels of blur. This study aimed to provide insight into the sensory modality of Milky Way compassing under a subset of realistic operational conditions through the use of a combination of established computer vision techniques and visualisations. We consider both the technical aspects of a solution and the implications of the solution on our understanding of the natural history of night-active insects. Firstly, we review what is known about insect vision in low light levels, their ability to resolve features, and examples of species where celestial sensitivity has been found. We then explore established motion blur models against this application, showing how point sources are affected compared to the MW. We demonstrate the effect on an example computer vision-based MW compass of motion blur on both simulated and real images. Finally, we examine the results and draw conclusions about why the MW might be useful to biological systems and why prospects may exist for its use in technological systems.

## 2. Background

The use of the MW for navigation is the result of evolutionary processes applied to particular insects in particular habitats. It is a technical solution to a survival problem and is intertwined with the fundamental limitations of their visual system and brain. We will consider here what is known, as it will be relevant to understanding how and where MW orientation is useful and where motion blur might fit within the solution.

### 2.1. Insect Vision

**Figure 2:**
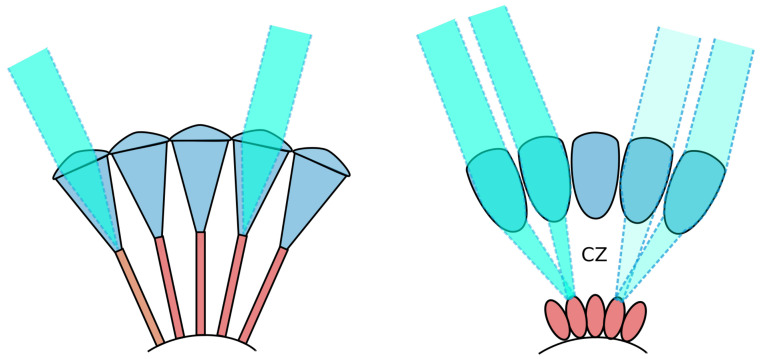
Simplified depiction of apposition (**Left**) and superposition (**Right**) compound eyes. The clear zone (CZ), found in superposition eyes, is labelled. Illustration adapted from [17].

Insect compound eyes are highly adaptable sensory structures [12]. Insects commonly possess one of two main types of compound eyes, apposition eyes and superposition eyes (Figure 2), each composed of cylindrical optical units known as ommatidia. In apposition eyes, typically found in diurnal insects like dragonflies [13] and honeybees [14], these ommatidia are sheathed in dark-coloured light-absorbing pigments that optically isolate them. This eye design allows a high spatial resolution but only limited sensitivity to light. Such eyes can thus resolve only the brightest stars [15] since the tiny front lens (i.e., facet) in each ommatidium drastically limits light capture. On the other hand, superposition compound eyes, which are common in nocturnal insects such as moths (e.g., the Elephant hawkmoth *Deilephila elpenor*) and beetles [11,16], are much more sensitive to light. Unlike in apposition eyes, in superposition eyes, the photoreceptors are withdrawn toward the back of the eye to create a wide optically transparent region, known as the clear zone (labelled CZ in Figure 2), between the lenses and the retina. Via specialised lens optics, a large number of facet lenses are recruited to collect and focus light across the clear zone and onto single photoreceptors in the retina, producing a very bright image but typically at the expense of spatial resolution. This lower resolution reduces the image sharpness of point sources, such as stars, but the much larger pupil typical of superposition eyes allows a significantly greater number of stars to become visible. In addition, due to the low F-number typical of superposition eyes, the extended MW will be seen with brilliant clarity.

Spatial pooling ensures that the effective angular resolution of the eye will be lower than that indicated by the density of the optical elements. This reduced spatial resolution renders the detection of individual stars by nocturnal insects unlikely. Given the already limited resolution of insect eyes compared to our own experience, the biological findings indicate that the MW may be a useful cue even for comparatively low-resolution vision systems.

### 2.2. The Celestial Compasses

Celestial bodies have been used by human navigators since ancient times. Even in the past few years, there is a sustained interest in the field of celestial navigation. Celestial objects, such as stars and the sun have continued to play an important role in land-based, maritime, and aerospace navigation [18,19,20,21,22,23,24]. Not only the information directly from the celestial object but also the celestial information like the polarised light from the sun or the moon can be used as an orientation cue in many navigation approaches and applications [25,26]. Some recent works have also achieved angle determination utilizing celestial information under low-light conditions that were inspired by biological navigation mechanisms; for example, one approach utilises a biomimetic polarisation sensor coupled with a fisheye lens to achieve night heading determination [27].

Among the remarkable navigation strategies exhibited by animals, celestial cues play an important role for orientation in both nocturnal and diurnal animals. Day-active insects, such as the honeybee [28,29,30] and the desert ant, *Cataglyphis* [3], use the sun and polarised light as compass cues. Three-quarters of a century ago, Karl Von Frisch used behavioural experiments to demonstrate that honeybees rely on celestial patterns of polarised light to navigate. When the angle of polarisation is changed, the honeybee changes its dance direction accordingly [31]. The desert ant uses the celestial polarisation pattern for path integration during foraging, thereby continuously maintaining a straight path back to its nest. To detect polarised light, insects possess specialised regions in the upward facing part of the compound eye (known as the dorsal rim area), which analyses skylight polarisation [32,33].

Compared with diurnal insects, nocturnal insects must maintain orientation precision under extremely dim light conditions. The illumination provided by a clear moonless night sky is significantly dimmer than full daylight, with a light intensity of around 0.0001 lux compared to 10,000 lux in daylight [34]. The pattern of celestial polarised light present around a full moon is up to a million times dimmer than that present around the sun during the day [35]. Nonetheless, when the moon is visible, celestial polarised moonlight can be used as an orientation cue. This circular pattern, centred on the moon, is caused by the atmospheric scattering of moonlight, just as sunlight is scattered during the day [36].

Some nocturnal insects use night celestial information for navigation and foraging. On moonlit nights, large yellow underwing moths (*Noctua pronuba*) navigate using the moon’s azimuth. When the moon is absent, behavioural experiments have shown that *N. pronuba* can orient using the celestial hemisphere [37]. Heart-and-dart moths (*Agrotis exclamationis*) also use the moon as a cue for orientation and appear to employ the geomagnetic field to calibrate their moon compass [38]. The nocturnal halictid bee, *Megalopta genalis*, with its specialised dorsal rim area capable of detecting the orientation of polarised light, may also use polarisation vision for navigation [39]. Moreover, the foraging behaviour of the nocturnal bee *Sphecodogastra texana* was observed to correlate with the lunar periodicity [40].

**Figure 3:**
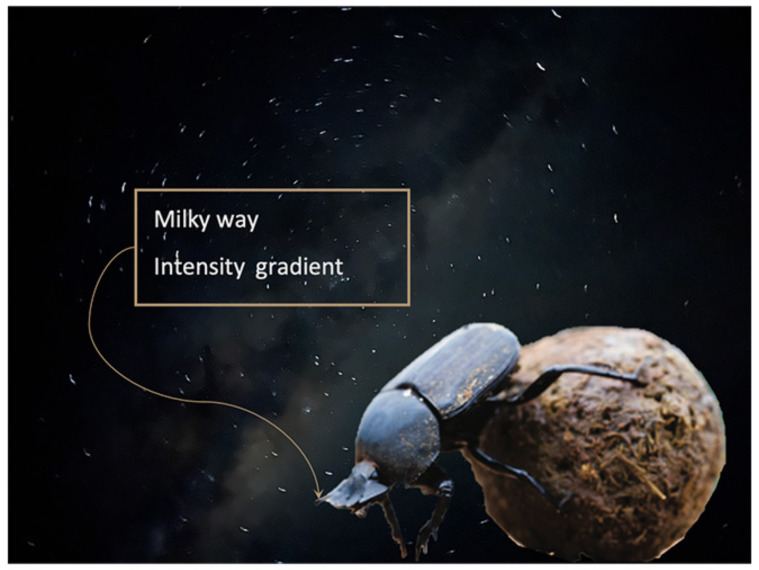
Representation of a nocturnal dung beetle in action under a moonless night sky.

When the moon and the lunar sky polarisation pattern are absent, nocturnal dung beetles *Scarabaeus satyrus* can use the MW for reliable orientation. These night-active dung beetles exhibit a robust orientation behaviour when only the MW is visible and use it as a stellar directional cue [41]. *S. satyrus* has the ability to navigate in very straight lines away from the dung pile while transporting the ball it has constructed away from competitors, despite a complex rolling task in the dark [10]. The locomotion of a dung beetle under a night sky is illustrated in Figure 3, showing why the rolling locomotion task is complex from a head stabilisation and navigation perspective.

The MW is a relatively bright extended streak arching across the dome of the night sky, providing a potential stellar orientation cue. Nocturnal insects can resolve only a few of the brightest individual stars, but integrated over its area, the MW is a bright and continuous object that is unambiguous in the night sky [11]. The MW orientation mechanism is based on a light intensity comparison between different regions of the MW [41]. The features of the MW that are used as orientation cues are its unique shape and gradient of increasing light intensity from the northern to the southern sky. Additionally, it is not likely to be impacted by minor changes in atmospheric conditions and is a reliable cue throughout the different seasons of the year.

It is important to understand the limitations of the approach used by dung beetles orienting at night. It seems that they are using the MW as a short-term heading reference, rather than as a compass that is aligned in some way to the inertial frame. In this regard, the mechanism could also be accurately described as a celestial landmark, although the distinction between a compass and a landmark is not significant given the time frame involved and the behaviour of the beetle.

The investigation of insect navigation not only unveils the fascinating adaptations of these tiny navigators but also provides valuable insights for biomimicry and the development of innovative navigation technologies. Studies inspired by insects that use celestial cues for navigation are an active research topic that has yielded profound insights, many of which have found their way into experimental robots and aircraft. Uses of celestial polarised light to achieve autonomous navigation both on the ground [42,43,44] and as part of a flying navigation system in a drone [45] have been developed and implemented. NASA has even considered the use of celestial polarisation for navigation in the challenging Mars environment, where the magnetic field is not useful for navigation and where deep terrain features surrounding the vehicle might mask the sun [46]. The use of the MW as a navigational cue for biomimetic robots has not, thus far, been demonstrated. The MW presents a different type of problem compared to the polarisation pattern, as it is resolvable without specialised optics, but a sophisticated computer vision system is required to identify and measure it, due to its low contrast, size, and shape.

### 2.3. Motion Blur in Navigation

Motion blur frequently manifests in real-world imaging scenarios. The impact of motion blur on performance has been widely acknowledged in the literature, including the significance of addressing motion blur as a pervasive issue that hinders the accurate interpretation of visual information in navigation tasks [47,48,49]. Motion blur in discretely sampled imaging systems is caused by movement across one frame of exposure; thus, long exposure times increase the magnitude of blur artefacts [50]. For a continuous imaging system such as those found in biological systems, the effect is created by movement comparable to the time constants of integration in the detectors. Motion blur effectively decreases resolution and causes coordinate errors for angular accuracy [48].

Extending exposure time is a way to capture sufficient light, but it simultaneously exacerbates the amount of motion blur. Under low-light conditions, consideration arises regarding the trade-off between long exposure with a strong signal and increased motion blur and short exposure with a low signal-to-noise ratio and high motion blur. Therefore, a balance between exposure duration and mitigating the motion blur effect in celestial navigation is needed to address the competing demands of gathering adequate light information and preserving celestial information in the captured imagery. Opportunities to reduce the effect of motion blur through reduced reliance on signals from point sources are useful, for example, by integrating across the angular size of a cue.

Despite insects having tiny brains and small compound eyes, the nocturnal dung beetle *Scarabaeus satyrus* can move in straight lines while it rolls its dung balls on a moonless night. The means of locomotion lends itself to extensive head and body movements and almost certainly to large angular motions of the head that will induce motion blur, as the rear pair of legs does the pushing, and the body is maintained at a high angle. Somehow, the use of the MW is possible despite this effect, or maybe the MW compass behavior has evolved because of this effect.

## 3. Data Collection

This section explains the data collection process. The MW is not visible in most urban centres across the world, either due to light pollution, atmospheric conditions, contaminants [51], or geographical location. Geographical location is relevant because the MW core is only visible in the south of the Northern Hemisphere; the brightest parts of the MW are generally not visible in high latitudes of the Northern Hemisphere. Compared with the Northern Hemisphere, the Southern Hemisphere is a better place for observing the MW. There is a “Milky Way season” from mid-autumn to mid-spring, during which the MW is most visible in Australia. During this time of the year, the centre or core of the MW Galaxy is clear to see when there is no moon or other light sources [52].

In this study, we used both real night sky images and synthesised night sky images to provide more data for a comprehensive analysis.

### 3.1. Real Data Acquisition

**Figure 4:**
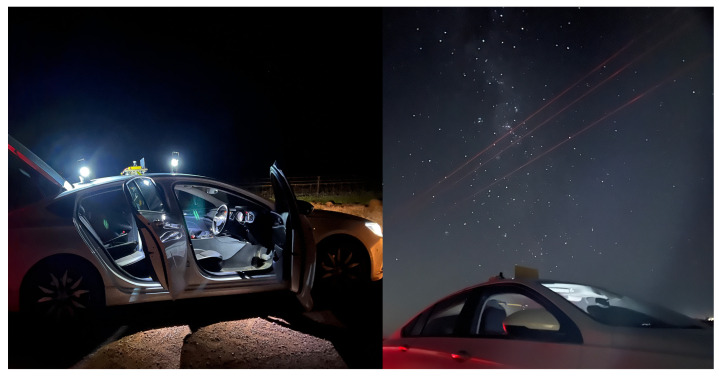
Vehicle-based data acquisition system, showing mounting and setup.

**Figure 5:**
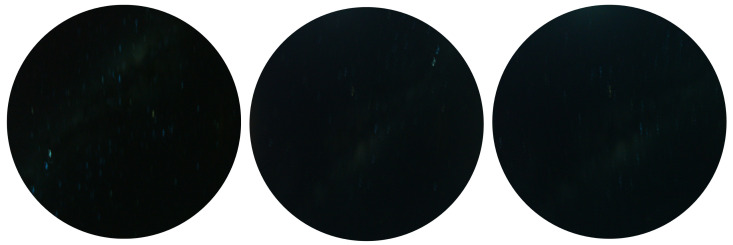
Exposure: 30 s, speed: 30–40 kph.

A data acquisition system, comprising a single-board computer (SBC) and standard OEM camera, was mounted to the roof of the test vehicle as shown in Figure 4. A night sky image dataset was then collected by driving around within the selected low-population-density region, located among the wheat fields surrounding Mallala, South Australia. The optical hardware used for capturing images was a Raspberry Pi HD camera with a 6 mm Wide Angle Camera Lens (CS-Mount). Initial imagery was captured while the vehicle was stationary but with the engine idling, and thus, the optical hardware was subject only to engine vibration. Once a sufficient collection of stationary imagery was obtained, we progressed to a non-stationary dataset, driving the vehicle at approximately 40 km/h, thus subjecting the optical hardware to both engine vibration (higher frequency) and road (lower frequency) vibration. The route taken by the vehicle contained a series of 90 degree bends and followed mostly coarse, unsealed roads. Additional images were captured with the vehicle stationary and engine switched off, providing reference data that were free from most significant sources of motion blur. Some additional information is provided in Table 1. All images in the dataset were captured in HD (3280×2464) format at 10 s, 20 s, and 30 s exposure times. The following images, Figure 5, were selected randomly and cropped to remove extraneous parts of the image. As we can see in Figure 5, the movements influence the data captured, and the motion blur occurred due to attitude changes in the vehicle and exposure time. In the celestial navigation of a moving insect, motion blur is likely to be substantial. Therefore, in the results section, we consider how the movement influences the visual orientation process.

### 3.2. Synthetic Data Generation

**Figure 6:**
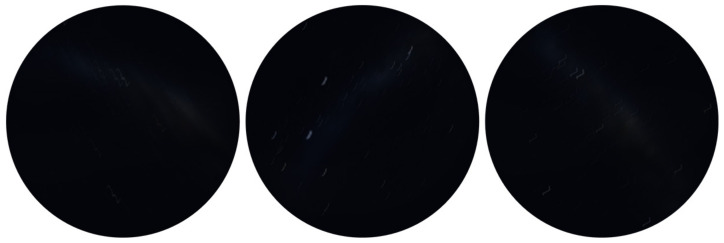
Synthesised night sky motion blur images.

The visibility of the MW in certain countries is hindered by light pollution. Also, it is a challenge to capture adequate night sky images with proper exposure, such that the MW is visible [53]. To overcome this limitation, Stellarium (version 0.22.2) was used to generate some MW images, and then the motion blur effects were added to create the synthesised night sky motion blur images, as shown in Figure 6.

**Figure 7:**
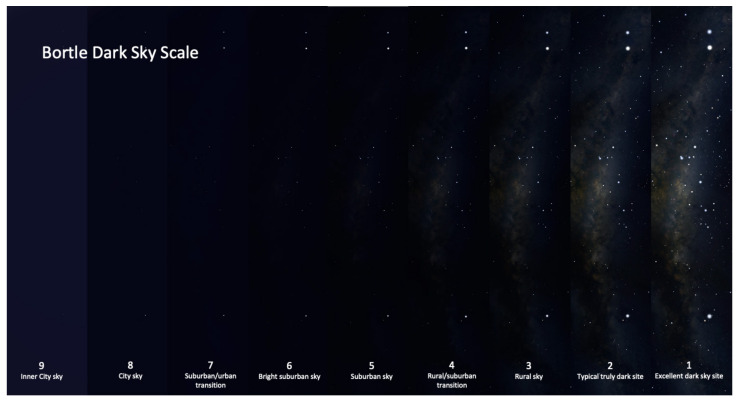
Bortle scale levels.

Stellarium is an open-source desktop planetarium software that was created for simulating the celestial sphere based on a given time and location [54]. There are several key settings in Stellarium that we used in this study: date, location, MW brightness/saturation, light pollution level (LP), etc. Figure 7 shows a part of the MW with different settings of the Bortle scale as the light pollution level in Stellarium. We selected three (rural sky) and four (rural/suburban transition) as the light pollution level settings [55]. All of the test images use the default MW brightness/saturation setting (brightness:1, saturation:1).

After the simulated night sky images were generated, we used the point-spread function (PSF)-based method to create synthesised night sky images with motion blur rendering [56]. In order to enhance the realism of synthesised images, the generation of diverse motion blur kernels contributes to a more authentic representation [56]. This method creates a motion blur kernel with a given intensity and size. There are several steps to generating the kernel:

- Calculate the maximum length ($Path_{max}$) of blur motion and the maximum angle ($Angle_{max}$) in radians;
- Calculate the length of the steps taken by the motion blur;
- Calculate a random angle for each step;
- The final step is to combine steps and angles into a sequence of increments and create a path out of these increments (Equations (1)–(4)):
  (1) $$Path_{max}=\left[Uni\left(0,1\right)+Uni\left(0,intensity^{2}\right)\right]\cdot diagonal\times 0.75$$
  (2) step=Beta(1,30)×(1−intensity+ϵ)×diagonal
  (3) $$Angle_{max}=Uni\left(0,intensity\times \pi \right)$$
  (4) $$angle=Tri\left(0,intensity\times Angle_{max},Angle_{max}+\epsilon \right)$$
  where Uni, Beta, and Tri are distribution functions used to generate random numbers [57]; diagonal is the length of the kernel diagonal; and the tiny error used for numerical stability is ϵ=0.1.

As we mentioned in Section 2.3, extending exposure time is a way to capture sufficient light, which has been used to capture clear MW images at night. As the exposure is relatively long, the details and features of the blur trails are significant. The synthesised images that we generated compared with the simulated image from Stellarium are characterised by traces and shapes made by the motion blur rather than points.

**Figure 8:**
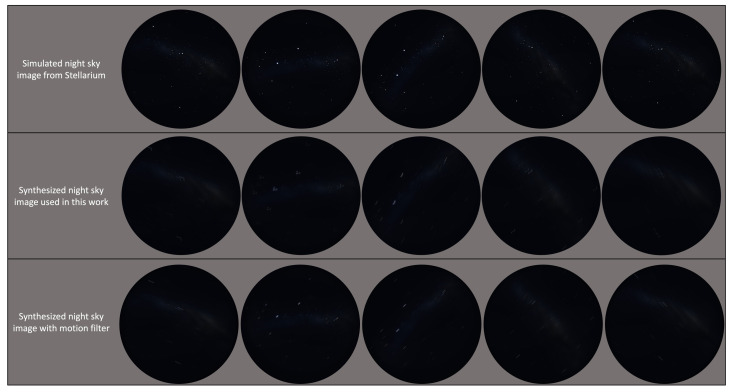
Comparison of the simulated images applied with the blur method used in this study and the motion blur effects with length and angle parameters (motion filter) [58].

**Figure 9:**
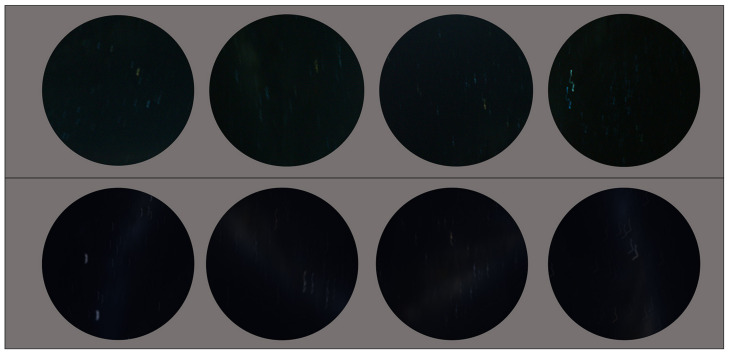
**Top row**: real night sky images. **Bottom row**: synthesised night sky images with motion blur effects.

Figure 8 demonstrates the different motion blur effects; the second row shows the motion blur method that we used in this study, which generates more realistic motion blur kernels. As we can see from Figure 9, the motion blur kernel in the real world (from a walking insect, aircraft, or insect in turbulence, or a vessel on a disturbed water surface) is not a simple line with a specific angle, which is commonly used in image processing to represent motion blur. The motion blur method we employed in this study can generate non-linear motion blur kernels with adjustable parameters, the intensity and size of the kernel. In Figure 9, to better observe the motion blur effects, we zoomed in on the real night sky images and synthesised night sky images with motion blur effects. The motion blur method we selected generates more realistic motion-blurred night sky images.

**Figure 10:**
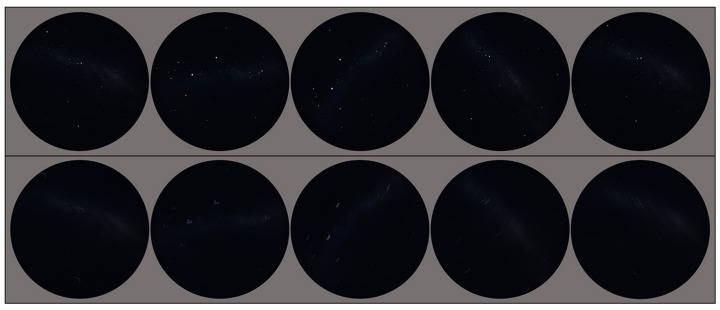
**Top row**: simulated night sky images from Stellarium. **Bottom row**: synthesised night sky images with motion blur effects.

Figure 10 shows a comparison of the simulated images and synthesised night sky images with motion blur effects.

## 4. Methodology

In previous work, we proposed a computer vision method that is capable of extracting direction information, under low light levels, of a large but low-contrast MW celestial landmark [59]. In this section, we provide a brief explanation of the method and extend the orientation estimation with a new method. The improved computer vision method used to detect the MW and find the orientation angle is presented in Algorithm 1. The MW orientation algorithm (MWOA) takes an RGB image and applies a series of image processing techniques, including noise removal and thresholding. The purpose of the algorithm is to expose the characteristics of the MW as an orientation landmark relevant to biomimetics, rather than to be a practical algorithm for autonomous navigation.    
**Algorithm 1:** MW Detection and Orientation  **Input**: RGB *image*  **Output**: *Angle***1** **for** *RGB image* **do****2** Split the image into its component red, green, and blue channels**3** Perform median filtering on each channel**4** Calculate the threshold level using Otsu’s method (in Section 4.1)**5** Convert the image into a binary image**6** Dilate image with a specified flat morphological structuring element**7** Remove small objects**8** Create output binary image**9** **for** *binary object mask image* **do****10** Calculate orientation using central moments (in Section 4.2)**11****return** *Angle*

### 4.1. Object Detection

The MW has been detected, and the mask is represented on a new binary image. First, Otsu’s image thresholding method [60] is calculated, and the spherical structuring element with a selected radius is used to generate the dilated binary object image. Then, objects containing fewer than a specified number of pixels are removed, and from this, the binary mask image is generated.

The following equations represent Otsu’s thresholding method of an image with *L* grey levels. Suppose we spilt the image pixels into two classes $C_{0}$ and $C_{1}$ using a threshold at level *t*. $n_{i}$ is the number of pixels with intensity *i*, and *N* is the total number of pixels. The probability of the occurrence of level *i* is given by $\frac{n_{i}}{N}$:

(5) $$p\left(i\right)=\frac{n_{i}}{N},p\left(i\right)\geq 0,\sum_{i}^{L-1}p\left(i\right)=1$$

The probabilities of two-class occurrence, respectively, are given by

(6) $$\omega_{0}=P\left(C_{0}\right)=\sum_{i=0}^{t}p_{i}=\omega \left(t\right)$$

(7) $$\omega_{1}=P\left(C_{1}\right)=\sum_{i=t+1}^{L-1}p_{i}=1-\omega \left(t\right)$$

The mean of the two classes is calculated based on the following equations:

(8) $$\mu_{0}=\sum_{i=0}^{t}\frac{i\cdot p(i)}{\omega_{0}}=\frac{1}{\omega (t)}\sum_{i=0}^{t}i\cdot p\left(i\right)$$

(9) $$\mu_{1}=\sum_{i=t+1}^{L-1}\frac{i\cdot p(i)}{\omega_{1}}=\frac{1}{1-\omega (t)}\sum_{i=t+1}^{L-1}i\cdot p\left(i\right)$$

The total mean can be written as

(10) $$\mu_{T}=\mu \left(L-1\right)=\sum_{i=0}^{L-1}ip_{i}$$

Otsu is defined as the between-class variance (BCV), which is shown as follows:

(11) $$\sigma_{B}^{2}=\omega_{0}{\left(\mu_{0}-\mu_{T}\right)}^{2}+\omega_{1}{\left(\mu_{1}-\mu_{T}\right)}^{2}$$

The optimal thresholding value of Otsu *t* is chosen by maximizing $\sigma_{B^{2}}$:

(12) $$t\;=\;\max\{\sigma_{B^{2}}\}$$

### 4.2. Orientation Estimation

Once the binary image has been generated I(x,y), the normalised second central moments for a region are used for calculating angular information for the extracted MW area. The centroid coordinates ($\bar{x},\bar{y}$) are calculated as the mean of the pixel coordinates in the *x* and *y* directions, respectively.

(13) $$\begin{array}{c} \bar{x}=\frac{1}{N}\sum_{i=1}^{N}x_{i} \end{array}$$

(14) $$\begin{array}{c} \bar{y}=\frac{1}{N}\sum_{i=1}^{N}y_{i} \end{array}$$

Here, *N* is the number of pixels in the region, and $x_{i}$ and $y_{i}$ are the *x* and *y* coordinates of the i−th pixel in the region, respectively.

Object orientation information can be derived using the second-order central moments, where central moment (μ) describes the coordinates of the mean. Normalised second central moments for the region ($\mu_{xx},\mu_{yy},\mu_{xy}$) can be calculated as follows:

(15) $$\mu_{xx}=\frac{\sum_{i}x_{i}^{2}}{N}+\frac{1}{12}$$

(16) $$\begin{array}{c} \mu_{yy}=\frac{\sum_{i}y_{i}^{2}}{N}+\frac{1}{12} \end{array}$$

(17) $$\mu_{xy}=\frac{\sum_{i}x_{i}y_{i}}{N}$$

where *N* is the number of pixels in the region, and *x* and *y* are the coordinates of the pixels in the region relative to the centroid. Also, $\frac{1}{12}$ is the normalised second central moment of a pixel with unit length:

(18) $$\begin{array}{cc} \; & \mathrm{num}=\left\{\begin{array}{cc} \mu_{yy}-\mu_{xx}+\sqrt{{\left(\mu_{yy}-\mu_{xx}\right)}^{2}+4\mu_{xy}^{2}}, & \;\mathrm{if}\;\mu_{yy}>\mu_{xx}\\ 2\mu_{xy}, & \;\mathrm{otherwise}\; \end{array}\right. \end{array}$$

(19) $$\begin{array}{cc} \; & \mathrm{den}=\left\{\begin{array}{cc} 2\mu_{xy}, & \;\mathrm{if}\;\mu_{yy}>\mu_{xx}\\ \mu_{xx}-\mu_{yy}+\sqrt{{\left(\mu_{xx}-\mu_{yy}\right)}^{2}+4\mu_{xy}^{2}}, & \;\mathrm{otherwise}\; \end{array}\right. \end{array}$$

The orientation of the region based on the normalised second central moments is as follows:

(20) $$\begin{array}{c} \;\mathrm{Orientation}\;=\frac{180}{\pi }\mathrm{atan}\left(\frac{\;\mathrm{num}\;}{\;\mathrm{den}\;}\right) \end{array}$$

## 5. Results

**Figure 11:**
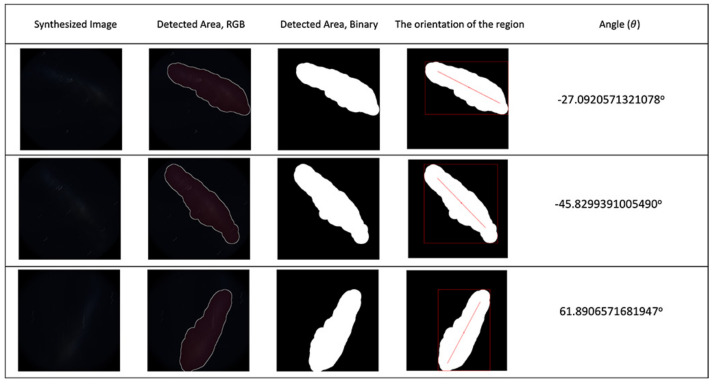
First column: synthesised night sky images; second to fifth columns: detection and angle calculation results for synthesised sky images. The light pollution level for the test images was 4.

The proposed technique was tested on both synthetic images and real night sky images. The synthetic test image results can be seen in Figure 11.

**Figure 12:**
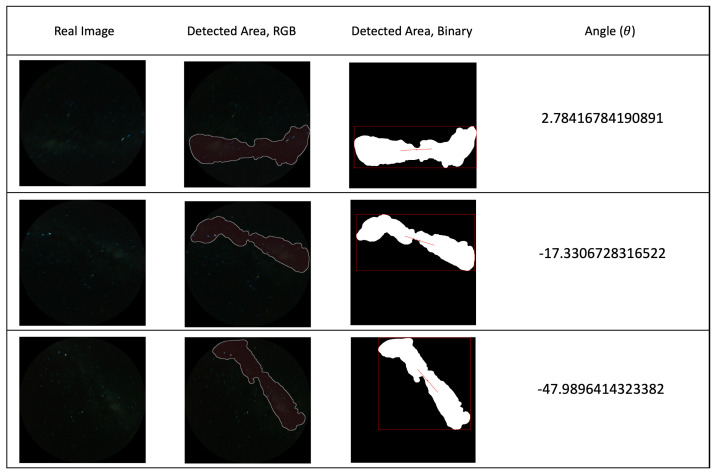
First column: real night sky images that are used for angle calculation test. second to fourth columns: detection and angle calculation results for real sky images (location: Mallala, South Australia; date: 12 July 2023; speed: 10 m/s; exposure time: 30 s).

The results from real night sky images are shown in Figure 12, which illustrates the MW detection and angle calculation results for the real night sky images.

**Figure 13:**
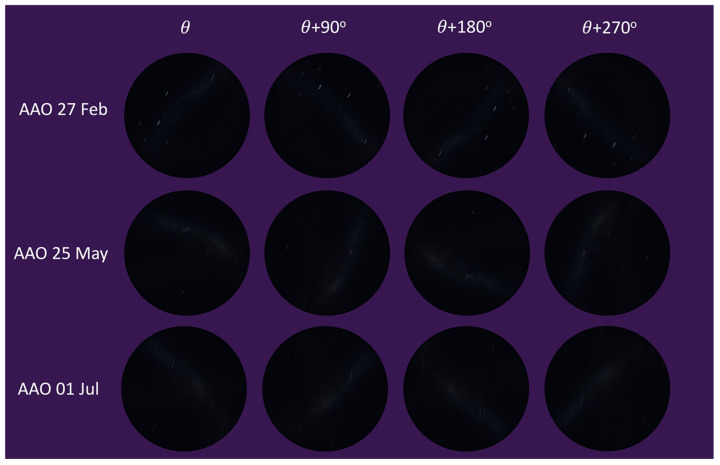
Moving angle calculation tested with synthetic images.

The intent of the algorithm (MWOA) is to use the MW as a celestial direction reference. The algorithm was executed on synthetic images that resulted from the observer undergoing a continuous rotation rate of 0.5°/s with updates at 10 s intervals, imposing both a rotation and the gradual movement of the celestial hemisphere and MW over time. All the dates and times for this test were set on different moonless nights. All of the images that were used for this angle calculation test were under LP:4 conditions (MW brightness:1 and saturation:1). Figure 13 shows a small subset of the test images from Stellarium with motion blur rendering for illustrative purposes. The images were captured from angle θ to θ+270° at 90° intervals.

The Radon transform (RT) has been used for extracting or reconstructing angular information in many image processing applications. The maximum value of the RT indicates the angle at which the highest intensity of the region corresponds to the orientation of the features in the image [61].

**Figure 14:**
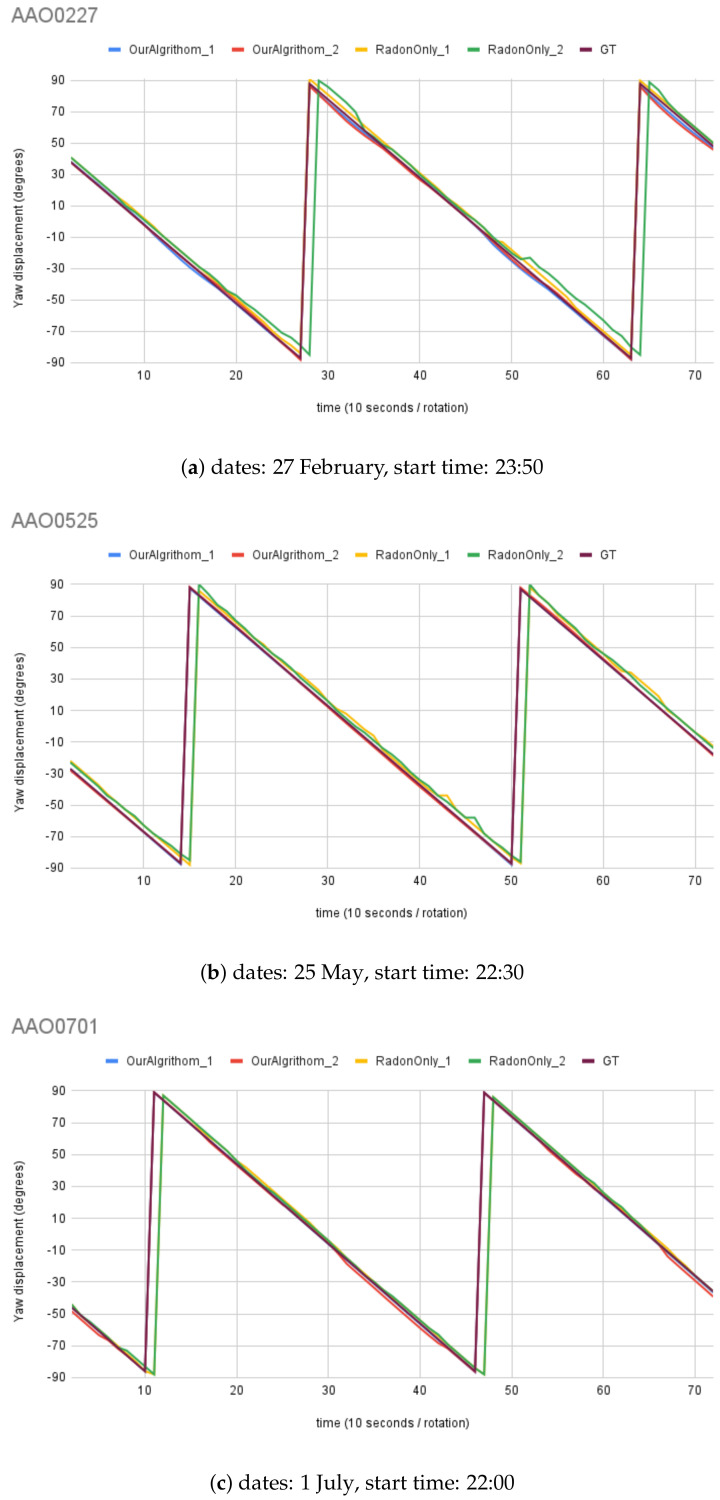
Moving angle calculation: location, Australia Astronomical Observatory (AAO).

**Figure 15:**
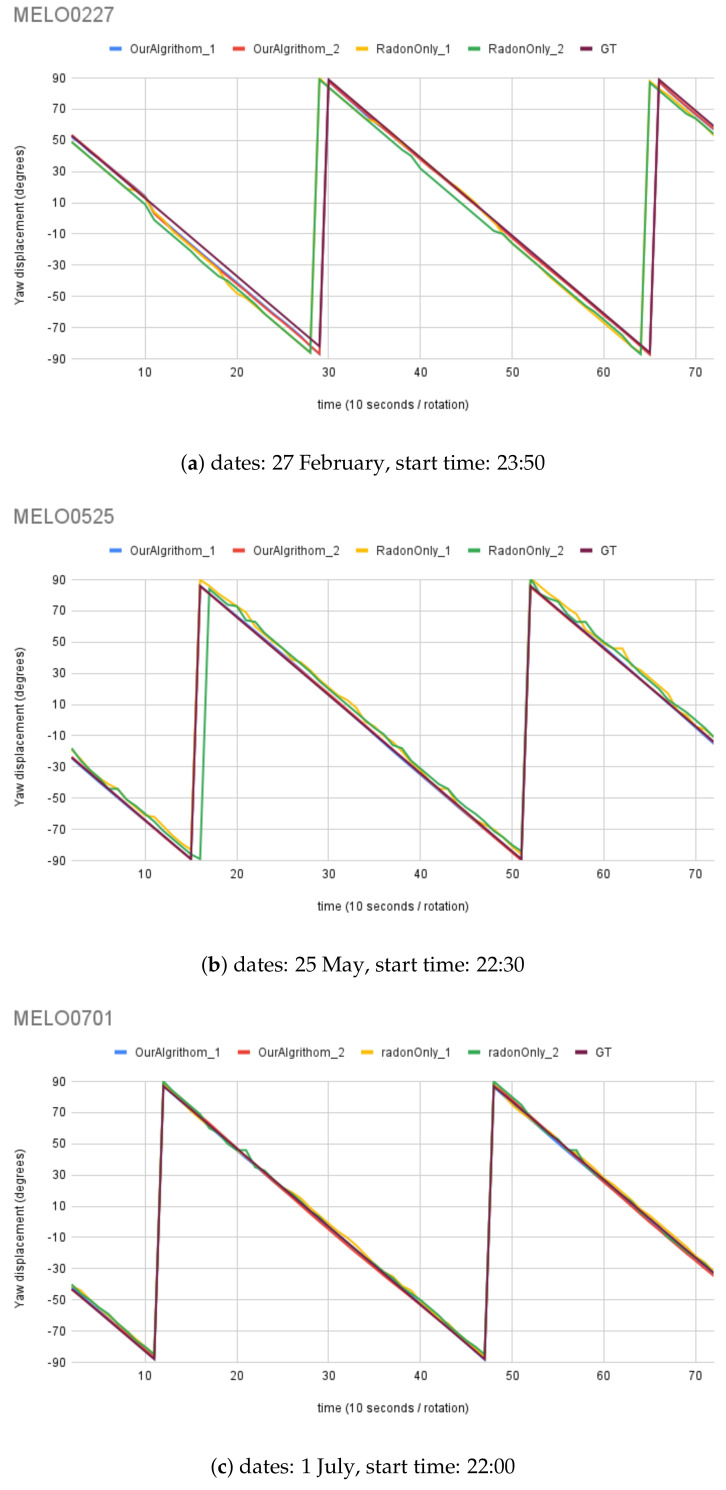
Moving angle calculation: location, Melbourne Observatory (MELO).

For synthetic images, the rotation angle calculation result comparison between the MWOA and RT are plotted in Figure 14 with the same observer location (Australia Astronomical Observatory, the AAO) on different dates. Figure 15 shows the results with another observer location (Melbourne Observatory, MELO). The dataset for each location contains six sets of synthetic images, totalling 432 images. For each date, two different random motion blur effects were applied to the same simulated image, and the results are shown in $RadonOnly_{1}$ and $RadonOnly_{2}$: $OurAlgorithm_{1}$ and $OurAlgorithm_{2}$, respectively. The Ground Truth (GT) angle is also shown with all the GT images starting from θ, which is set as the starting angle from OurAlgrithom. As we can see in Figure 14b, the blue line ($OurAlgorithm_{1}$) and the red line ($OurAlgorithm_{2}$) closely follow the moving angle displacement (5° interval rotations), as shown in the purple line (GT) compared to the yellow line ($RadonOnly_{1}$) and the green line ($RadonOnly_{2}$), showing that the MWOA provides a more reliable orientation calculation in the presence of motion blur compared with the pure Radon transform algorithm. Because we applied two different motion blur kernels in each test set, the RT-only algorithm results—the yellow line ($RadonOnly_{1}$) and the green line ($RadonOnly_{2}$)—show quite different results for some test images, as shown in Figure 14a and Figure 15b. By comparison, the orientation angle results for the MWOA—the blue line ($OurAlgorithm_{1}$) and the red line ($OurAlgorithm_{2}$)—are highly overlapped, which indicates the reliability of our method on different blur images. The error is measured and summarised in Table 2 and Table 3 for different observer location test sets, the AAO and MELO respectively.

**Figure 16:**
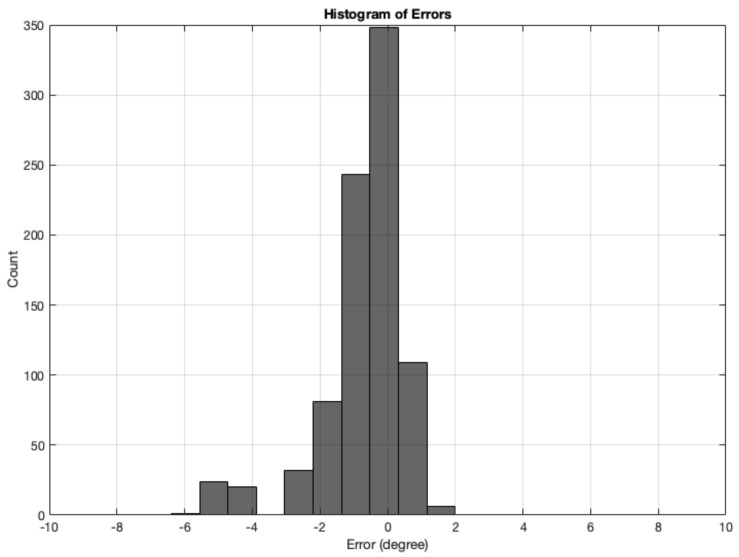
Histogram of errors between the angle calculation and the GT angle using the MWOA for all synthetic images.

The error is measured as the difference between the angle calculation tested with synthetic images and the Ground Truth angle. The range of ground truth is 0–180, with the GT starting from θ, which is set as the starting angle from $OurAlgrithom_{1}$. The simulated images dataset for angle calculation contains 12 test sets of synthetic images, totalling 864 images. The results from all synthetic image angle result errors are summarised in Table 4. The mean absolute error was 0.95833°. Figure 16 shows a histogram of the angle errors.

**Figure 17:**
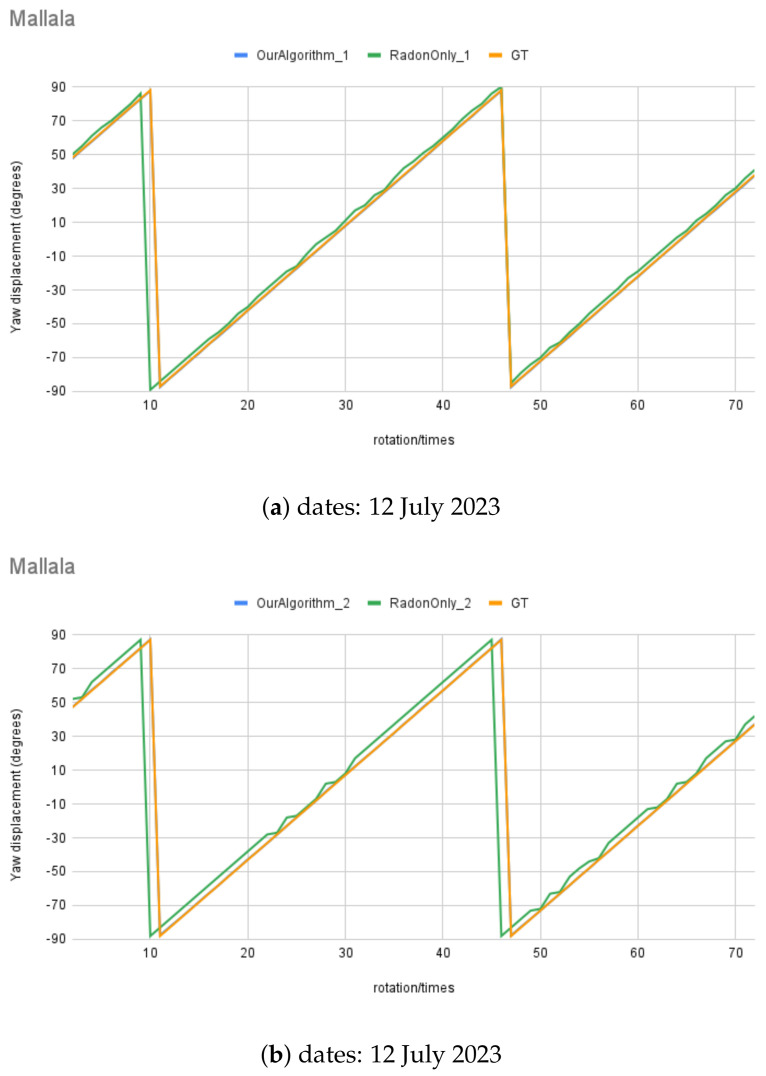

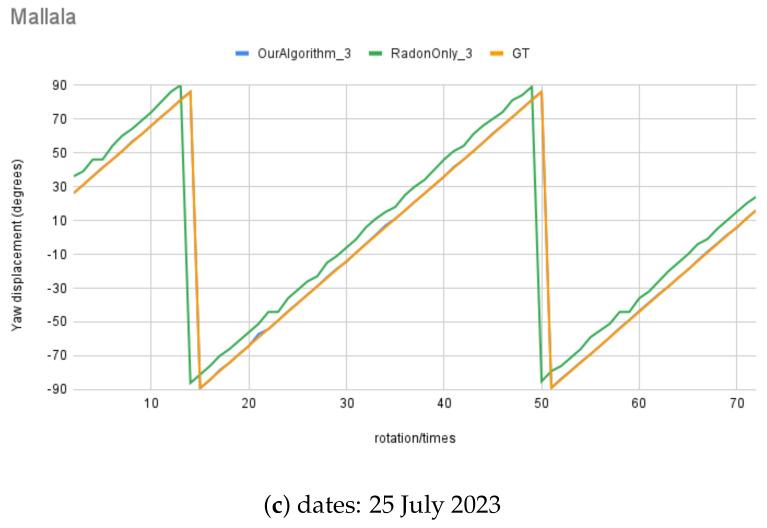
Moving angle calculation of real night sky image captured in Mallala, South Australia.

**Figure 18:**
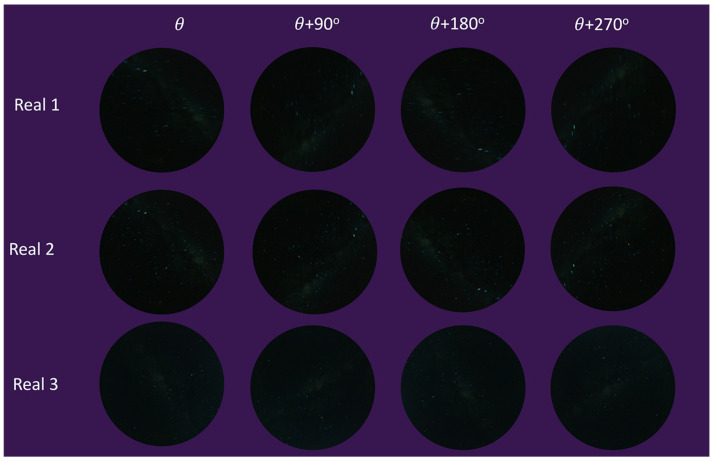
Moving angle calculation with real test images.

Also, Figure 17 illustrates the real night sky images’ rotation angle calculation results for the three selected night sky images. The rotation angle calculation test with real images was conducted using an imposed rotation rate of 5° each step; in total, there were 72 test images for each test set, and some images are shown in Figure 18, at angle θ to θ+270°, at 90° intervals. The RT (RadonOnly) was also tested with comparable results, shown in comparison to the MWOA ($OurAlgorithm_{1}$) for real images.

**Figure 19:**
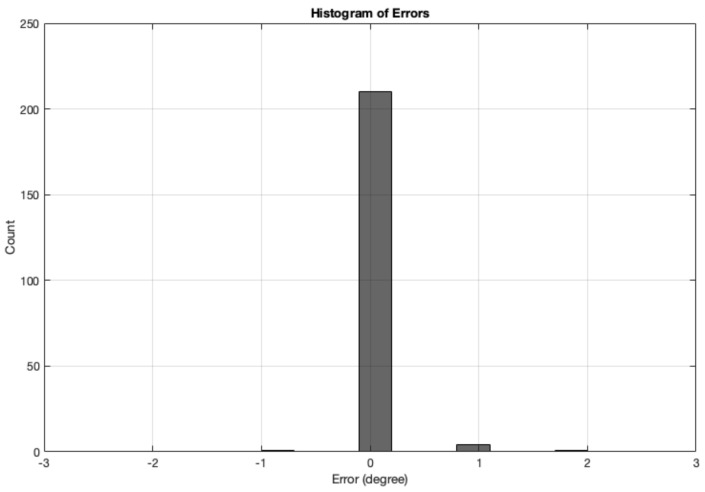
Histogram of errors between the angle calculation and the GT angle using the MWOA for all real images.

The error is measured as the difference between the angle calculation tested with real images and the Ground Truth angle. The range of ground truth is 0–180, with the GT starting from θ, which was set as the starting angle from $OurAlgrithom_{1}$. The error results from all real-image (totalling 216 images) angle calculation are summarised in Table 5. The mean absolute error was 0.032407. Figure 19 shows a histogram of the angle errors.

## 6. Discussion

Figure 14, Figure 15, and Figure 17 illustrate the performance of the MWOA when the motion blur was applied. As discussed in Section 5, all the test images were digitally rotated 5° each time step, for a total of 72 images for each test set. Therefore, the Ground Truth of the orientation calculation for the test images can be calculated from the starting angle (θ), which is then ±5° (depending on the rotation direction) for each rotation. The mean absolute error for each test set of synthetic test images angle is between a minimum of 0.22° and a maximum of 1.61° for the MWOA, while the mean absolute error is between 0.60° and 4.68° for the RT-only algorithm. In addition, for real night sky images, the mean absolute error is between a minimum of 0.00° and a maximum 0.08° for the MWOA, compared with the RT-only algorithm, which is between a minimum of 0.72° and a maximum of 1.90° for each test set. The performance of the method we used of extracting the MW shape as the orientation cue appears to be more accurate and reliable under real-world circumstances.

As the magnitude of blur increases, the individual star intensities drop until they are barely visible on the screen or printed image. It is clear that at some level of motion blur, the signal of individual blurred stars will fall below the sensor noise.

By contrast, the MW is a more resilient target for position and orientation. The results show that the motion blur from real images undergoing real motion on a vehicle, as well as synthetic images, has minimal effect on the calculation of angles, using the example orientation measurement method.

For the dung beetle pushing its payload facing backward, while under a moonless sky, the amount of movement of the head and body is likely to be substantial. Under these circumstances, we have shown that the MW is a robust orientation landmark. The individual star intensity would be substantially diminished under these circumstances.

With regard to orientation accuracy, rather than the detection of sky orientation, the primary articulation of the insect head is anatomically in the body frame roll and pitch axes, with less motion possible in the yaw. Bearings taken from individual stars would be strongly affected in the case of substantial roll and pitch, since the rotations would result in large apparent shifts in the projection of the star onto the eye. The effect on the orientation of the MW as an entire shape, by comparison, is substantially less if the insect’s visual system can detect the orientation of the MW as an object.

Applications of a nocturnal orientation sensor may include small ground robots operating under the same conditions as nocturnal beetles. The likelihood of clouds is an obvious environmental limit at low altitudes on Earth. More exotic applications might include drones, orbiting satellites, both in deep space and on other planets in the solar system. The resistance of the MW cue to motion blur, and the large area over which orientation calculation can be undertaken, may make the cue useful for automatic orientation in low-light conditions.

## 7. Conclusions

In this paper, we demonstrated that the measurement of the orientation of the Milky Way celestial landmark, using optical hardware, is robust to motion blur that might be caused by rotational vibration and stabilisation artefacts. We also showed, through motion blur filters applied to real and synthetic images, that the imaging of the Milky Way is largely unaffected by blur. When exposed to the same motion blur, even the brightest star’s intensity is dramatically reduced, making stars much less useful as celestial landmarks. The proposed techniques were tested and validated using a diverse dataset generated from synthetic data and acquired field data. Future work will focus on learning more about low-resolution celestial navigation challenges and solutions in real-time flying robot implementations.

## Figures and Tables

**Table 1:** Information about the vehicle data acquisition system used at Mallala, South Australia.

Item	Specification
Location	Mallala, South Australia, Australia
Moving speed	0–40 kmh
Camera	Pi HD camera
Exposure	10–30 s
Computer	Raspberry Pi 3B+

**Table 2:** Errors between the angle calculation using the MWOA and the Ground Truth (AAO).

AAO Synthetic-Image Angle Errors			
	**Mean Error**	**Median**	**Standard Deviation**
Simulated angle errors (degree)	−0.55324	0	1.0843
Absolute simulated angle errors (degree)	0.67824	0	1.0095

**Table 3:** Errors between the angle calculation using the MWOA and the Ground Truth (MELO).

MELO Synthetic-Image Angle Errors			
	**Mean Error**	**Median**	**Standard Deviation**
Simulated angle errors (degree)	−0.80093	−1	1.5239
Absolute simulated angle errors (degree)	1.1759	1	1.2552

**Table 4:** Errors between the angle calculation using the MWOA and the GT angle.

All Synthetic-Image Angle Errors			
	**Mean Error**	**Median**	**Standard Deviation**
Simulated angle errors (degree)	−0.67824	0	1.3387
Absolute simulated angle errors (degree)	0.95833	1	1.1539

**Table 5:** Errors between the angle calculation and the Ground Truth using the MWOA.

All Real-Image Angle Errors			
	**Mean Error**	**Median**	**Standard Deviation**
Simulated angle errors (degree)	0.023148	0	0.20328
Absolute simulated angle errors (degree)	0.032407	0	0.20154

## Data Availability

The original contributions presented in the study are included in the article and supplementary material, further inquiries can be directed to the corresponding author/s.

## Abbreviations

The following abbreviations are used in this manuscript:

MW	Milky Way;
LP	light pollution;
BCV	between-class variance;
MWOA	MW orientation algorithm;
RT	Radon transform;
MELO	Melbourne Observatory;
AAO	Australia Astronomical Observatory;
SBC	single-board computer
GT	Ground Truth.
